# The World Health Organization's World No Tobacco Day 2020 Campaign Exposes Tobacco and Related Industry Tactics to Manipulate Children and Young People and Hook a New Generation of Users

**DOI:** 10.1016/j.jadohealth.2020.06.026

**Published:** 2020-09

**Authors:** Simone St Claire, Ranti Fayokun, Alison Commar, Kerstin Schotte, Vinayak M. Prasad

**Affiliations:** Department of Health Promotion, World Health Organization, Geneva, Switzerland

There are 1.3 billion tobacco users worldwide. That number might be even larger if tobacco did not kill half of its users [1]. Every 4 seconds, tobacco is responsible for another premature death [2]. For decades, the tobacco industry has deliberately used aggressive, duplicitous, and well-resourced tactics to hook generations of users to nicotine and tobacco, driving the global tobacco epidemic. This is primarily achieved through engineering and manipulating of products to sustain addiction, with young people being the main target. The strategy is to replace smokers and ensure market sustainability by making the products appealing and attractive to new and existing users, especially youth. In 1984, R.J. Reynolds stated, “younger adult smokers are the only source of replacement smokers… If younger adults turn away from smoking, the industry must decline” [3]. Such strategies to engage children and adolescents before they are fully aware of the ramifications of their actions have been successfully used by the industry since the 1970s and are still in use today.

Over the last decade, as the awareness of the harms of tobacco use has grown and global tobacco control efforts have intensified, the social acceptability of tobacco use has declined, directly impacting the sale of the most popular product—the cigarette. To maintain its profitability, the multi-billion-dollar industry has aggressively started to look for newer markets in low- and middle-income countries and also come up with innovative and creative ways to stay relevant and to keep its products on the market. Thus, it has been trying to reinvent itself by introducing a new portfolio of products, presenting itself as part of the solution to combat the tobacco epidemic, which it created in the first place. The strong marketing and promotional strategy of the tobacco industry has led to an increase in nicotine and tobacco product use among youth globally [[4], [5], [6], [7], [8], [9], [10]].

An overview of the three major categories of portfolio products and their health risks are summarized below:

1. **Conventional tobacco products, such as cigarettes, smokeless tobacco, and waterpipe tobacco**
Cigarette smoking is the most common form of tobacco use worldwide. Other tobacco products include waterpipe tobacco (commonly known as shisha or hookah), various smokeless tobacco products (chewing tobacco, snus, and snuff), cigars, cigarillos, roll-your-own tobacco, pipe tobacco, bidis, and kreteks.
  The tobacco epidemic is one of the biggest public health challenges the world has ever faced, killing more than eight million people globally every year [2]. Nearly all tobacco use begins in childhood and adolescence, and early onset provides more life-years to tobacco use, increasing the risk of developing tobacco-related diseases [11]. Tobacco use among youth increases the risk of reduced lung function, impaired lung growth, and early onset of chronic respiratory disease [11]. Starting to smoke in childhood doubles the risk of premature death [12]. The lungs continue to grow well into adulthood, but inhaling the toxic substances of tobacco smoke slows this process and causes potentially irreversible lung damage [11]. Tobacco is deadly in any form, whether smoked or unsmoked. Tobacco smoke contains more than 7000 chemicals, including some known to cause cancer and the use of smokeless tobacco products has been linked to health problems, and sometimes death [13]. Furthermore, second-hand smoke has been linked to several adverse health outcomes, including death [14].

2. **Heated tobacco products**
Heated tobacco products (HTPs) are like all other tobacco products—inherently toxic, contain carcinogens, and expose users to toxic emissions, many of which cause cancer.
  HTPs are tobacco products that produce aerosols containing the highly addictive substance, nicotine, and toxic chemicals, some of which are carcinogens (i.e., substances that can cause cancer in humans) which is inhaled by the user [15]. Currently, there is no evidence to demonstrate that HTPs are less harmful than conventional tobacco products, and they contain chemicals not found in cigarette smoke, which may have associated health effects [15].

3. **E-cigarettes**
Electronic nicotine delivery systems and electronic non-nicotine delivery systems, more commonly referred to as e-cigarettes, are devices that heat a liquid to create an aerosol that is inhaled by the user. E-cigarettes do not contain tobacco but typically contain nicotine and toxic substances that are harmful to health [16,17].
  In combination with tobacco smoking, which is the practice of the majority of e-cigarette users, the health effects of two or more products are combined [18]. Evidence on the harmful health effects of e-cigarettes is mounting, as their use has been associated with heart disease [19] and lung disorders [20]. They pose significant risks to pregnant women who use them, as they can damage the growing fetus [16].
  E-cigarettes are particularly dangerous when used by children and adolescents. Nicotine is highly addictive and can have long-lasting, damaging effects on brain development [21,22]. Furthermore, there is a growing body of evidence in some settings that never-smoker minors who use e-cigarettes at least double their chance of starting to smoke conventional tobacco cigarettes later in life [23]. In addition, exposure of children to the liquid contained in e-cigarettes could pose serious risks to children if they leak or children swallow the liquid [24]. Some e-cigarettes have also been reported to cause serious injuries, including burns, through fires and explosions [24].

## Decades of Deception and Manipulation to Keep People Hooked to Addictive Products

Using manipulative marketing tactics and social positioning techniques, the industry has effectively targeted children and adolescents with this expanded portfolio of products that threaten their future [25]. Tobacco and related industries have a well worked out strategy and calculated approach to expand their market share with existing and newer products and also to expand to new markets, especially in low- and middle-income countries [25,26].

The tobacco industry has a long-standing history of misleading the public about the risks associated with other tobacco products. Between the 1950s and 1970s, the industry introduced cigarette filters and “light” and “mild” cigarettes as evidence mounted around the harms of tobacco, which it promoted as an alternative to quitting, while being fully aware that those products were not less harmful to health [13]. Today, the industry continues misleading the public by suggesting that some tobacco products are less harmful than others before the body of evidence on the harms of these products can be fully established.

## Tobacco and Related Industry Strategies to Increase Adolescent Use of Nicotine and Tobacco Products Globally

Traditional forms of advertising, promotion, and sponsorship by the tobacco industry are well-known billboards, radio and television advertisements, point-of-sale displays, brand sharing, brand stretching, and event sponsorship (Table 1). However, the industry is continuing to look at new opportunities aimed at making nicotine and tobacco products appealing, sustaining use. This is with the primary objective of maximizing profit irrespective of the harms caused by these products, especially to young people.

**Table 1:** Tobacco and related industry strategies to increase adolescent use of nicotine and tobacco products globally

Advertising	**Digital and social media advertising:** Tobacco and related industries have strategically used digital and social media platforms to reach younger generations [25], including through their favorite apps and video games [27,28]. **Product placement in entertainment media, such as television and cinema** [29]**:** Children and adolescents who watch movies and television shows containing depictions of smoking are at an increased risk of initiating smoking. In 2018, at least half of the movies with depictions of tobacco were youth-rated [30], and the number of depictions of tobacco in youth-rated films has grown 63% since 2015 [31].
Promotion	**Free product samples and merchandise:** The tobacco industry also promotes their products by distributing free samples and merchandise with tobacco logos. In more than 50 countries, at least 10% of students aged 13–15 years reported ever being offered a free cigarette by a tobacco company representative. In more than 120 countries, at least 1 in 10 students aged 13–15 years reported having an object with a tobacco company logo [32].
Sponsorship	**Scholarships and school programs:** Tobacco and e-cigarette-related entities have offered scholarships and paid schools for the opportunity to speak in classrooms or after school [33,34]. They have also sponsored summer camps to spread misconceptions about the risks of e-cigarette use and market their products under the guise of promoting “safer alternatives” to conventional tobacco products [35]. **Celebrity and influencer endorsements:** “Influencers” on social media who reach and engage children and adolescents are invited by these industries to serve as “brand ambassadors” or offered financial incentives to promote their tobacco products [36,37].
Other marketing tactics	**Flavors that appeal to youth:** The tobacco industry has made tobacco products, such as smokeless and waterpipe tobacco, more palatable by marketing them in sweet and fruity flavors, which increase appeal and mask the harsh tobacco taste [38,39]. E-liquid flavors are available in a variety of flavors, including those proven to appeal to youth, such as cotton candy and gummy bear [40]. **Sleek designs:** E-cigarettes and heated tobacco products are extensively promoted as modern, high-tech and high-end lifestyle products, with minimalist designs, and high-profile product launches that portray them as attractive and harmless products. **Single stick cigarettes and disposable e-cigarettes:** Products have been made more affordable to young people through the sale of single stick cigarettes [41] and disposable e-cigarettes [42], which typically lack health warnings.

## World No Tobacco Day 2020

The World No Tobacco Day 2020 campaign serves to debunk myths and expose devious tactics used by tobacco and related industries to attract children and young adults. It also raises awareness about the common and covert tactics used by these industries and provides young people with the knowledge required to easily detect and stand up against industry manipulation. This campaign reinforces the World Health Organization's work in assisting country-level implementation of effective policy interventions to reduce the demand for tobacco, thereby promoting well-being and ensuring maximum protection of public health and overall, strengthening tobacco control globally.

The World Health Organization calls on all young people to join the fight to become a tobacco-free generation. The world cannot afford another generation deceived and exploited by the lies and subterfuge of the tobacco industry, which pretends to promote freedom of personal choice while really pursuing eternal profits–profits that cost millions of lives every year. (https://www.who.int/news-room/campaigns/world-no-tobacco-day/world-no-tobacco-day-2020).
